# COMMUNITY-LEVEL SOCIAL CAPITAL AND ALL-CAUSE MORTALITY IN JAPAN: FINDINGS FROM THE ADACHI COHORT STUDY

**DOI:** 10.1093/geroni/igae098.2870

**Published:** 2024-12-31

**Authors:** Hiroshi Murayama, Mika Sugiyama, Hiroki Inagaki, Chiaki Ura, Fumiko Miyamae, Ayako Edahiro, Tsuyoshi Okamura, Shuichi Awata

**Affiliations:** Tokyo Metropolitan Institute for Geriatrics and Gerontology, Tokyo, Japan; Tokyo Metropolitan Institute for Geriatrics and Gerontology, Tokyo, Japan; Tokyo Metropolitan Institute for Geriatrics and Gerontology, Tokyo, Japan; Tokyo Metropolitan Institute for Geriatrics and Gerontology, Tokyo, Japan; Tokyo Metropolitan Institute for Geriatrics and Gerontology, Tokyo, Japan; Tokyo Metropolitan Institute for Geriatrics and Gerontology, Tokyo, Japan; Tokyo Metropolitan Institute for Geriatrics and Gerontology, Tokyo, Japan; Tokyo Metropolitan Institute for Geriatrics and Gerontology, Tokyo, Japan

## Abstract

Community-level social capital is known to be associated with various health outcomes, but its impact on mortality is not fully understood. This study examined the association between community-level social capital and all-cause mortality among community-dwelling older Japanese people. The baseline data was obtained from a questionnaire survey for all 132,005 residents aged ≥65 years without long-term care insurance certification in Adachi Ward of the Tokyo metropolitan area in 2015 (75,358 valid responses; mean age 73.8 years; 45.0% males). We measured two aspects of social capital: neighborhood cohesion (trust, attachment, and a sense of belonging) and neighborhood network (neighborly ties). We stratified these into high, medium, and low categories. For community-level social capital, we calculated the proportion of those with high and medium neighborhood cohesion and neighborhood network by 262 administrative areas in Adachi. The study protocol was approved by the IRB. A sex-stratified multilevel survival analysis (average follow-up of 1,656 days), after adjusting for both individual-level and community-level covariates, showed that community-level neighborhood cohesion was negatively associated with all-cause mortality in men (hazard ratios: 0.92 [0.84-1.00] for the highest quintile and 0.91 [0.82-0.99] for the second, compared to the lowest), but not in women. These suggest that for men, who typically have fewer social networks and support systems than women, the benefits of living in a cohesive community may be life-extending. This supports the notion that interventions aimed at enhancing community social capital could be a strategic component in public health efforts to reduce mortality risks, particularly among older men.

